# Respiratory Syncytial Virus Infection Modeled in Aging Cotton Rats (*Sigmodon hispidus*) and Mice (*Mus musculus*)

**DOI:** 10.1155/2022/8637545

**Published:** 2022-03-09

**Authors:** Olivia E. Harder, Stefan Niewiesk

**Affiliations:** Department of Veterinary Biosciences, College of Veterinary Medicine, the Ohio State University, Columbus, OH, USA

## Abstract

Serious infection with respiratory syncytial virus (RSV) is associated with high risk in infants, children, and elderly. There is currently no approved vaccine against RSV infection, and the only available prevention is immunoprophylaxis utilized in high-risk infants, leaving the elderly without many options. In the elderly, the chronic low-grade inflammatory state of the body can play a significant role during infection. The cotton rat and mouse have emerged as the preferred small animal models to study RSV infection in the elderly. These animal models of aging have shown an age-dependent time course for clearance of virus correlating with a significantly diminished cytotoxic T lymphocyte and humoral immune response in old animals compared to adult animals. In addition, protection through vaccination is reduced in aging rodents. These results mirror the findings in humans. In mice and cotton rats, treatment with ibuprofen, a nonselective nonsteroidal anti-inflammatory drug (NSAID), to decrease the chronic low-grade inflammation of the elderly immune system has proven successful in restoring the function of cytotoxic lymphocytes. While more research is required, these treatment types promise a beneficial effect in addition to a putative vaccine. Choosing an appropriate animal model to study RSV infection in the aging immune system is essential to benefit the growing population of elderly in the world. This review focuses on the current research of RSV infection in the cotton rat and mouse as model systems for an aging immune system.

## 1. Introduction

### 1.1. Respiratory Syncytial Virus

Respiratory syncytial virus (RSV) is a nonsegmented negative sense enveloped RNA virus. RSV previously belonged to the *Paramyxoviridae* family, but was reclassified into the Pneumoviridae family in 2016 [1]. The risk for RSV infection is increased by a history of asthma, exposure to smoke, and other chronic diseases [2–4]. Disease burden is high for infants and the elderly. RSV infection is responsible for 14,000 deaths annually and about 200,000 hospitalizations among the adult population over 65 years of age in the United States [5, 6]. Because there is no RSV vaccine, numerous vaccine platforms are being used to develop RSV vaccines for adults and high-risk groups including the elderly [7]. The single prophylaxis for RSV is palivizumab (Synagis®), a neutralizing humanized monoclonal antibody specific for the F (fusion) protein of RSV. Palivizumab is licensed by the Food and Drug Administration for application in preterm infants and infants with a congenital heart disease for the prevention of severe RSV lower respiratory tract infections [8]. The treatment is costly and reserved for infants with high risk of severe disease. Subsequently, affordable treatments for RSV infection in the elderly would be an attractive complement to vaccination.

### 1.2. Aging Immune System

Understanding the aging immune response of the elderly is essential for the development of new therapeutics and vaccines. It has been shown that the aging of the immune response (immunosenescence) is characterized by reduction in antigen recognition and processing by dendritic cells and effector function of T cell subsets [9]. It has also been suggested that the increasing, continuous low-level systemic inflammation in old age is responsible for increased disease development. Currently, the aging body is seen as a constitutive proinflammatory environment (inflammaging) with continuous low-grade innate immune activation that may increase tissue damage caused by infections in the elderly [10]. Inflammaging is a key parameter of virtually every major age-related disease and has been shown to be a defining pathological characteristic of aging tissues across multiple species [11]. It has been suggested that elevated baseline inflammation may hamper the response of T cells and B cells to antigenic stimulation [12]. This aging and chronic low-grade inflammatory state of the elderly immune system can play a significant role during infection.

Virus-induced damage and host response to RSV infection both contribute to the development of RSV disease, and age, which affects host immune status, is a major determinant of RSV disease severity and prognosis [13]. In the elderly, RSV infection is after influenza virus infection, the second most common cause for viral pneumonia and death [14]. Influenza and RSV infections both follow similar seasonal trends and peak in the winter months of December through February. Although influenza virus infection is causing higher mortality rates, these can theoretically be prevented by vaccination [15]. However, even with an approved vaccine, vaccination rates among the elderly are below 70% for the past 10 years [16]. Also, the effectiveness of the influenza vaccine in the elderly was below 62% for any one subtype over the past 5 seasons [17]. The low vaccine effectiveness and vaccination rates against influenza in the elderly can be detrimental. In seniors, declining immune responses leads to a decreased efficacy of vaccines [18]. It has also been shown that in the elderly, defective B cell [19, 20] and T cell [21] functions are the major players in the decreased protection of the influenza vaccine. Also, it has been shown that inflammation-related genes were negatively correlated with influenza-specific antibody responses, supporting the concept that inflammatory responses at baseline might be detrimental to vaccine-induced antibody responses [22]. The development of a vaccine for RSV directed towards the elderly needs to consider the declining immune function in this age group. To date, preclinical development for RSV vaccines is typically performed in young seronegative animals [23], thus modeling humans with a fully functional immune system. However, healthy adults with a functioning mature immune system generally develop effective anti-RSV immunity [24]. In order to align vaccines with the requirements of an aging immune system, the immune response in old animals must be understood and vaccinations need to be tested in these animals.

## 2. Animal Models for RSV

The selection of an animal model depends heavily upon the type of RSV disease being studied. There is no single animal model which replicates all aspects of primary RSV disease in the human adult or the at-risk subpopulations [25]. The calf, cotton rat, mouse, guinea pig, ferret, and nonhuman primates (chimpanzee, owl monkey, rhesus macaque, African green monkey, Cebus monkey, squirrel monkey, bonnet monkey, and baboon) have all been utilized in some ways for RSV research. However, the cotton rat (*Sigmodon hispidus*) and mouse (*Mus musculus*) have been utilized as the preferred small animal models for investigating RSV infection in the elderly population [26].

### 2.1. The Cotton Rat and Mouse as Models for RSV Infection in an Aging Immune System

The cotton rat and mouse have proven to be valuable animal models for studying RSV in the presence of an aging immune system. From the age of eighteen months and older, mice show significantly increased susceptibility to RSV infection, increased lung histology, and reduced immunity [23, 27–29], which is similar to the situation in humans. Also, similar to the situation in elderly humans, from the age of eight months and older, cotton rats show significantly increased susceptibility to RSV infection and reduced immunity [30–33]. The mouse model has several advantages over all other species, including a vast array of inbred, congenic, transgenic, and knockout strains; an unmatched library of specific reagents allowing identification and quantification of cell types, immunoglobulins, cytokines, and other antigens; and relatively low purchase and maintenance costs [25, 34, 35]. One of the biggest advantages to using cotton rats to study RSV is their lung anatomy. In both human and cotton rat bronchioles [36], the lining consists of ciliated, columnar respiratory epithelial cells [37]. As ciliated, columnar respiratory epithelial cells are targets for RSV infection, and virus infected cells are found in both the upper and lower respiratory tract in the cotton rat; whereas in mice, only lung tissue is infected [38]. Cotton rats and mice do not develop clinical signs of disease after RSV infection, although cotton rats produce a 100-fold more virus than BALB/c mice [39]. A difference to the mouse is the scarcity of immunological reagents available for cotton rats, although cotton rat-specific reagents for all basic immunological techniques exist [40, 41]. With these advantages for both models, numerous studies have focused on the aging immune system and its correlation with RSV infection. These studies are focused on viral replication, vaccination, and potential treatment options for the elderly.

## 3. What We Have Learned from Both Models

### 3.1. Viral Replication

One of the biggest unresolved questions of RSV infection in the elderly is why the virus disproportionately affects this age group over others. In humans, it is known that clearance of RSV infection depends on the age of the infected individual [2]. It is necessary to have an animal model that also shows this age-dependent change in the clearance of virus. In both the cotton rat and mouse, aging animals take 5 days longer to clear virus in their lungs when compared to naïve, adult animals. When comparing adult (6 weeks and older), naïve cotton rats to the aging cotton rats (8 months and older) at 6 days postinfection with RSV, aging rats had significantly greater viral titers in their lungs and noses [32, 42]. Aging cotton rats also have a delayed clearance of virus, taking up to 9 days postinfection with RSV to completely clear virus from their lung and nose when an adult animal clears in 6 days [33]. Aging mice were shown to also have a delayed clearance of virus, taking up to 12 days to completely clear virus from their lungs, whereas an adult animal clears in 7 days [27, 43]. Older mice also experienced greater weight loss and more severe bronchiolitis and increased number of inflammatory cells in alveolar spaces via lung histology [29]. Clearly, both models show an age-dependent change in the clearance of virus. Further investigation into the immune response of these aging animals could shed light on why this is the case.

### 3.2. Immune Response

#### 3.2.1. Innate

As the first line of defense against pathogens, the innate immune response is essential for the recruitment of immune cells to the site of infection. Key players of the innate immune response include natural killer cells, macrophages, neutrophils, and dendritic cells. Functions of macrophages, natural killer cells, neutrophils, and dendritic cells were reported to decline with age [44]. A delay in the production of IFN-ɣ was observed in aging cotton rats, with expression levels peaking on day 6 for aging animals compared to day 4 for young animals [30]. An age-related decline of natural killer cells, an important source of IFN-ɣ, could explain the decrease in RSV-induced IFN-ɣ in older animals. Overall, this delay in RSV-induced cytokine expression was attributed to an age-related defect in the innate immune response. Another key player in the innate immune response, the expression of macrophage Toll-like receptors (TLRs), is reduced with age [45]. It has been shown that TLR4 plays an essential role in regulating innate immune response to RSV [46]. Peripheral blood mononuclear cells (PBMCs) from old individuals (65 years) have been shown to have a delayed and altered transcriptional response to stimulation with TLR4 [47]. In cotton rats, aging animals had a delayed expression of IL-10 and MCP-1 [30]. It is possible that reduced responsiveness of macrophages in the aging cotton rats, associated with decreased expression of TLR4, might result in delayed production of cytokines, such as IL-10 and MCP-1 during RSV infection. These cytokines are essential for recruitment of immune cells to the site of infection and delayed and/or decreased production of cytokines could lead to a diminished adaptive immune response.

#### 3.2.2. Adaptive

The reduction in the frequency of naïve T cells together with the increasing proportion of terminally differentiated T lymphocytes have long been hallmarks of immune aging [48]. Also, it has been shown that deficiencies in RSV-specific T cell responses lead to severe RSV disease in the elderly [49]. As in humans, the importance of RSV-specific T cells and how these subsets change during aging has also been investigated in these aging animal models. It is known that cytotoxic lymphocytes and the adaptive immune response are essential for suppressing RSV infection in cotton rats [50]. The depletion of cytotoxic lymphocytes (CD8 T cells) during RSV infection in aging cotton rats led to a further delay in viral clearance, indicating the important role of CD8 T cells in defense against RSV infection [33]. Also, it was found that age-related changes of the adaptive immune system of the cotton rat were apparent in reduced frequencies of T cells in both the spleen and lung in response to RSV [31]. In the mouse, aging mice mount diminished number of RSV-specific CD8 T cells at the peak magnitude of the acute T cell response [27, 28]. Decreased or diminished RSV-specific CD8 T cell responses in the aging population can contribute to disease severity. Altogether, current research points to the necessity of an appropriate CD8 T cell response in order to reduce disease severity in the elderly.

While the importance of cytotoxic T lymphocytes during RSV infection has been shown, the humoral immune response can also play a key role in acute infection and protection against reinfection as well. In the aging body, the numbers of naïve B cells decrease and effector B cells accumulate leading to a reduction in the diversity of antibody responses [51]. A loss of diversity in the B cell repertoire would have dramatic and serious consequences for the integrity of the humoral immune system [52]. Also, the boost in serum antibody following natural RSV reinfection in elderly adults appears to be short lived [53]. It has also been shown in the cotton rat that age-related changes to the immune system can lead to an impaired capacity to mount antibodies in response to RSV [31, 33, 42]. These studies demonstrated that aging cotton rats had significantly lower neutralizing and total RSV-specific antibody levels when compared to adult cotton rats. It was also shown that aging cotton rats produce significantly fewer RSV-specific antibody secreting B cells in their lung draining lymph node, spleen, and bone marrow when compared to an adult cotton rat [33]. Aging mice have also shown a reduced antibody response to RSV when compared to young mice. Aging mice had significantly lower neutralizing antibody titer when compared to young mice after immunization with RSV [23]. It is clear in both aging animal models and humans that there is also an age-dependent reduction in B cells in response to RSV.

One of the biggest hurdles of an aging immune system is vaccination. In humans, the reduced capacity of both innate and adaptive immune responses is thought to contribute to the decreased efficacy of vaccines in the elderly [18]. Simply put, deficient immune responses lead to inadequate vaccination. In adult, naïve cotton rats, vaccination with RSV leads to complete protection from challenge. It has been shown that while vaccination with RSV provided some protection, aging cotton rats still produced detectable virus in their lung and nose upon challenge [33]. Also, it has been shown that aging cotton rats have an age-dependent reduction of the ability to mount protective immunity to RSV challenge, in both primary and secondary (booster) immune responses [42]. The mouse has also proven to be a valuable model when investigating vaccine candidates. When testing vaccine candidates already in human clinical trials, researchers found that two fusion protein-based subunit vaccine candidates were not able to induce a completely protective immune response in RSV-seronegative aging mice at the evaluated vaccine dose [23]. The aging mice benefitted from the addition of an adjuvant (GLA-SE) during immunization but were still unable to mount a protective immune response. These results agreed with the human clinical trial results, further proving the need for appropriate animal models when testing vaccine candidates. Altogether, the elderly population could benefit from preclinical vaccine trials in age-appropriate animal models.

### 3.3. Treatment Options

As the immune response to RSV infection in the elderly continues to be investigated, treatment options have emerged, while vaccines are still being developed. With the knowledge of the chronic low-grade inflammatory state of the elderly immune system, anti-inflammatory treatments have become increasingly popular. The use of antiviral (palivizumab) and anti-inflammatory (corticosteroid) treatments have been previously tested in adult, naïve cotton rats. Treatment with palivizumab alone leads to faster viral clearance, but little improvement in pulmonary lesions. By combining antiviral and anti-inflammatory drugs, reversal of pulmonary histopathology is caused by RSV, while, at the same time, overcoming the immunosuppressive effect of the glucocorticosteroid was achieved [54]. With the promising results in young animals, the same treatment was tested in aging cotton rats [30]. While this treatment was successful in suppressing RSV replication in the lungs of aging cotton rats, it also led to increased mortality in this age group.

In mice, it was shown that there is an age-related increase in prostaglandin D_2_ (PGD_2_) which correlates with a progressive impairment in respiratory dendritic cells (rDC) migration to draining lymph nodes. Decreased rDC migration resulted in diminished T cell responses and more severe clinical disease in older mice infected with respiratory viruses (SARS-CoV and IAV-infected mice) [55]. Other treatment options have also been developed utilizing the aging cotton rats pointing to prostaglandins and cyclooxygenase 2 (COX-2) as potential targets for RSV therapy. It has been shown that COX-2, a precursor to various prostaglandins, is induced by RSV infection [56, 57]. The physiologic effects of prostaglandins (i.e., inflammatory processes) have been suggested to be important factors in the RSV disease process. Treatment of adult, naïve cotton rats with a COX-2 inhibitor significantly mitigated lung histopathology produced by RSV [56]. With the knowledge of increased prostaglandin and COX-2 expression, the use of nonsteroidal anti-inflammatory ibuprofen, a nonselective COX inhibitor, has been investigated to test their benefits during RSV infection in aging cotton rats [33]. In this study, the ibuprofen treatment leads to clearance of virus in geriatric cotton rats 3 days sooner, reducing infection from 9 to 6 days, which is similar to a young, naive adult cotton rat. Also, if vaccinated during ibuprofen treatment, the geriatric cotton rats were completely protected from challenge. When determining the role of ibuprofen in the clearance of virus, this study indicated that the ibuprofen treatment restored the functionality of cytotoxic CD8 T lymphocytes in geriatric cotton rats. Depletion of cytotoxic lymphocytes during ibuprofen treatment completely abolished the treatment effect seen in the geriatric cotton rats.

## 4. Conclusion

In a society where the aging population continues to grow, understanding the aging immune system is crucial. The cotton rat and mouse models have been invaluable in researching inflammation, adjuvants, and vaccines. Utilizing the cotton rat and mouse as animal models of the aging immune system during RSV infection has proven successful, while more research studies are necessary. Investigating new treatment options and testing vaccine candidates in these age appropriate models is essential to maintain the health of the growing population of elderly.

## References

[1] Lefkowitz E. J., Dempsey D. M., Hendrickson R. C., Orton R. J., Siddell S. G., Smith D. B. (2018). Virus taxonomy: the database of the international committee on taxonomy of viruses (ICTV). *Nucleic Acids Research*.

[2] Hall C. B., Simőes E. A. F., Anderson L. J. (2013). Clinical and epidemiologic features of respiratory syncytial virus. *Current Topics in Microbiology and Immunology*.

[3] Walsh E. E., Peterson D. R., Kalkanoglu A. E., Lee F. E.-H., Falsey A. R. (2013). Viral shedding and immune responses to respiratory syncytial virus infection in older adults. *The Journal of Infectious Diseases*.

[4] P P., M S., J P. (2021). *Underlying Cardiopulmonary Conditions as a Risk Factor for Influenza and Respiratory Syncytial Virus Infection Among Community-Dwelling Adults Aged ≥ 65 Years in Thailand: Findings from a Two-Year Prospective Cohort Study*.

[5] Falsey A. R., Hennessey P. A., Formica M. A., Cox C., Walsh E. E. (2005). Respiratory syncytial virus infection in elderly and high-risk adults. *New England Journal of Medicine*.

[6] Matias G., Taylor R., Haguinet F., Schuck-Paim C., Lustig R., Shinde V. (2017). Estimates of hospitalization attributable to influenza and RSV in the US during 1997-2009, by age and risk status. *BMC Public Health*.

[7] Higgins D., Trujillo C., Keech C. (2016). Advances in RSV vaccine research and development - a global agenda. *Vaccine*.

[8] Cardenas S., Auais A., Piedimonte G. (2005). Palivizumab in the prophylaxis of respiratory syncytial virus infection. *Expert Review of Anti-infective Therapy*.

[9] Heppner H. J., Cornel S., Peter W., Philipp B., Katrin S. (2013). Infections in the elderly. *Critical Care Clinics*.

[10] Shaw A. C., Joshi S., Greenwood H., Panda A., Lord J. M. (2010). Aging of the innate immune system. *Current Opinion in Immunology*.

[11] Finch C. E., Morgan T. E., Longo V. D., de Magalhaes J. P. (2010). Cell resilience in species life spans: a link to inflammation?. *Aging Cell*.

[12] Frasca D., Blomberg B. B. (2016). Inflammaging decreases adaptive and innate immune responses in mice and humans. *Biogerontology*.

[13] Wen X., Mo S., Chen S. (2019). Pathogenic difference of respiratory syncytial virus infection in cotton rats of different ages. *Microbial Pathogenesis*.

[14] A W., Tma W. (2021). Respiratory viral infections in the elderly. *Therapeutic Advances in Respiratory Disease*.

[15] Thompson W. W., Shay D. K., Weintraub E. (2003). Mortality associated with influenza and respiratory syncytial virus in the United States. *JAMA*.

[16] (2020). https://www.cdc.gov/flu/fluvaxview/coverage-1718estimates.htm.

[17] Russell K., Chung J. R., Monto A. S. (2018). Influenza vaccine effectiveness in older adults compared with younger adults over five seasons. *Vaccine*.

[18] Weinberger B., Herndler‐Brandstetter D., Schwanninger A., Weiskopf D., Grubeck‐Loebenstein B. (2008). Biology of immune responses to vaccines in elderly persons. *Clinical Infectious Diseases*.

[19] Frasca D., Diaz A., Romero M. (2010). Intrinsic defects in B cell response to seasonal influenza vaccination in elderly humans. *Vaccine*.

[20] X Y., A J L., S I. (2020). Antibody and local cytokine response to respiratory syncytial virus infection in community-dwelling older adults. *mSphere*.

[21] Saurwein-Teissl M., Lung T. L., Marx F. (2002). Lack of antibody production following immunization in old age: association with CD8+CD28− T cell clonal expansions and an imbalance in the production of Th1 and Th2 cytokines. *The Journal of Immunology*.

[22] Nakaya H. I., Hagan T., Duraisingham S. S. (2015). Systems analysis of immunity to influenza vaccination across multiple years and in diverse populations reveals shared molecular signatures. *Immunity*.

[23] Cayatte C., Snell Bennett A., Rajani G. M. (2017). Inferior immunogenicity and efficacy of respiratory syncytial virus fusion protein-based subunit vaccine candidates in aged versus young mice. *PLoS One*.

[24] B B, Je C., A V (2015). Effect of preexisting serum and mucosal antibody on experimental respiratory syncytial virus (RSV) challenge and infection of adults. *The Journal of Infectious Diseases*.

[25] Byrd L. G., Prince G. A. (1997). Animal models of respiratory syncytial virus infection. *Clinical Infectious Diseases*.

[26] Rudd P. A., Chen W., Mahalingam S. (2016). Mouse and cotton rat models of human respiratory syncytial virus. *Human Respiratory Syncytial Virus, *.

[27] Fulton R. B., Weiss K. A., Pewe L. L., Harty J. T., Varga S. M. (2013). Aged mice exhibit a severely diminished CD8 T cell response following respiratory syncytial virus infection. *Journal of Virology*.

[28] Zhang Y., Wang Y., Gilmore X., Xu K., Wyde P. R., Mbawuike I. N. (2002). An aged mouse model for RSV infection and diminished CD8+ CTL responses. *Experimental Biology and Medicine*.

[29] Zhan L., Zhao M., Yi H. (2016). [Comparison of respiratory syncytial virus infection on different week-ages BALB/c mice]. *Bing du xue bao = Chinese J Virol.*.

[30] Boukhvalova M. S., Yim K. C., Kuhn K. H. (2007). Age‐related differences in pulmonary cytokine response to respiratory syncytial virus infection: modulation by anti‐inflammatory and antiviral treatment. *The Journal of Infectious Diseases*.

[31] Guichelaar T., van Erp E. A., Hoeboer J. (2018). Diversity of aging of the immune system classified in the cotton rat (*Sigmodon hispidus*) model of human infectious diseases. *Developmental & Comparative Immunology*.

[32] Curtis S. J., Ottolini M. G., Porter D. D., Prince G. A. (2002). Age-dependent replication of respiratory syncytial virus in the cotton rat. *Experimental Biology and Medicine*.

[33] Harder O. E., Martinez M., Niewiesk S. (2021). Nonsteroidal anti-inflammatory drugs restore immune function to respiratory syncytial virus in geriatric cotton rats (*Sigmodon hispidus*). *Virology*.

[34] Domachowske J. B., Bonville C. A., Rosenberg H. F. (2004). Animal models for studying respiratory syncytial virus infection and its long term effects on lung function. *The Pediatric Infectious Disease Journal*.

[35] Kong X., Hellermann G. R., Patton G. (2005). An immunocompromised BALB/c mouse model for respiratory syncytial virus infection. *Virology Journal*.

[36] Zhang L., Peeples M. E., Boucher R. C., Collins P. L., Pickles R. J. (2002). Respiratory syncytial virus infection of human airway epithelial cells is polarized, specific to ciliated cells, and without obvious cytopathology. *Journal of Virology*.

[37] Grieves J. L., Yin Z., Durbin R. K., Durbin J. E. (2015). Acute and chronic airway disease after human respiratory syncytial virus infection in cotton rats (*Sigmodon hispidus*). *Comparative Medicine*.

[38] Irvin C. G., Bates J. H. (2003). Measuring the lung function in the mouse: the challenge of size. *Respiratory Research*.

[39] Taylor G. (2017). Animal models of respiratory syncytial virus infection. *Vaccine*.

[40] Niewiesk S., Prince G. (2002). Diversifying animal models: the use of hispid cotton rats (*Sigmodon hispidus*) in infectious diseases. *Laboratory Animals*.

[41] Green M. G., Huey D., Niewiesk S. (2013). The cotton rat (*Sigmodon hispidus*) as an animal model for respiratory tract infections with human pathogens. *Lab Animal*.

[42] Guichelaar T., Hoeboer J., Widjojoatmodjo M. N. (2014). Impaired immune response to vaccination against infection with human respiratory syncytial virus at advanced age. *Journal of Virology*.

[43] Liu B., Kimura Y. (2007). Local immune response to respiratory syncytial virus infection is diminished in senescence-accelerated mice. *Journal of General Virology*.

[44] Plackett T. P., Boehmer E. D., Faunce D. E., Kovacs E. J. (2004). Aging and innate immune cells. *Journal of Leukocyte Biology*.

[45] Renshaw M., Rockwell J., Engleman C., Gewirtz A., Katz J., Sambhara S. (2002). Cutting edge: impaired toll-like receptor expression and function in aging. *The Journal of Immunology*.

[46] Haynes L. M., Moore D. D., Kurt-Jones E. A., Finberg R. W., Anderson L. J., Tripp R. A. (2001). Involvement of toll-like receptor 4 in innate immunity to respiratory syncytial virus. *Journal of Virology*.

[47] Metcalf T. U., Cubas R. A., Ghneim K. (2015). Global analyses revealed age‐related alterations in innate immune responses after stimulation of pathogen recognition receptors. *Aging Cell*.

[48] Nasi M., Troiano L., Lugli E. (2006). Thymic output and functionality of the IL-7/IL-7 receptor system in centenarians: implications for the neolymphogenesis at the limit of human life. *Aging Cell*.

[49] Cherukuri A., Patton K., Gasser R. A. (2013). Adults 65 years old and older have reduced numbers of functional memory T cells to respiratory syncytial virus fusion protein. *Clinical and Vaccine Immunology*.

[50] Wethington D., Harder O., Uppulury K. (2019). Mathematical modelling identifies the role of adaptive immunity as a key controller of respiratory syncytial virus in cotton rats. *Journal of The Royal Society Interface*.

[51] Allman D., Miller J. P. (2005). B cell development and receptor diversity during aging. *Current Opinion in Immunology*.

[52] Gibson K. L., Wu Y.-C., Barnett Y. (2009). B-cell diversity decreases in old age and is correlated with poor health status. *Aging Cell*.

[53] Falsey A. R., Singh H. K., E E. W. (2006). Serum antibody decay in adults following natural respiratory syncytial virus infection. *Journal of Medical Virology*.

[54] Prince G. A., Mathews A., Curtis S. J., Porter D. D. (2000). Treatment of respiratory syncytial virus bronchiolitis and pneumonia in a cotton rat model with systemically administered monoclonal antibody (palivizumab) and glucocorticosteroid. *The Journal of Infectious Diseases*.

[55] Zhao J., Zhao J., Legge K., Perlman S. (2011). Age-related increases in PGD 2 expression impair respiratory DC migration, resulting in diminished T cell responses upon respiratory virus infection in mice. *Journal of Clinical Investigation*.

[56] Richardson J. Y., Ottolini M. G., Pletneva L. (2005). Respiratory syncytial virus (RSV) infection induces cyclooxygenase 2: a potential target for RSV therapy. *The Journal of Immunology*.

[57] Za R., Dk M., M R A. (2010). Pulmonary cyclooxygenase-1 (COX-1) and COX-2 cellular expression and distribution after respiratory syncytial virus and parainfluenza virus infection. *Viral Immunology*.

